# Dural Venous Sinus Thrombosis: A Rare Cause of Intracranial Hemorrhage

**DOI:** 10.7759/cureus.73693

**Published:** 2024-11-14

**Authors:** Harris Alam, Dru Curtis, Syed A Aftab

**Affiliations:** 1 Radiology, Florida State University College of Medicine, Orlando, USA; 2 Radiology, AdventHealth Orlando, Orlando, USA

**Keywords:** dural vein, head trauma, mr venography, pediatric head injury, sinus thrombosis

## Abstract

Dural venous sinus thrombosis, a subset of cerebral venous thrombosis, is an important pathology due to its significant morbidity and mortality. This process has an annual incidence of three to five cases per million adults. Although copious predisposing factors exist, the core principles revolve around Virchow’s triad: venous stasis, hypercoagulability, and vascular endothelial damage. There are various risk factors related to unfavorable outcomes, which include female sex, intracranial hemorrhage, infection, cancer, and more. Trauma tends to be a risk factor, particularly in pediatric populations, as in this patient’s case. Initial diagnosis in the acute setting commonly involves non-contrast CT studies to exclude hemorrhage. In subacute or chronic settings, MRI can be utilized. Anticoagulation remains the mainstay of therapy for treatment, along with treating any underlying causes of thrombosis. We present a pediatric patient with extensive post-traumatic dural venous sinus thromboses with classic imaging findings as well as full resolution of clinical symptoms after this pathologic process was recognized and treated appropriately.

## Introduction

Dural venous sinuses are endothelial-lined venous channels within the cerebral venous network that help drain blood from the brain's superficial structures. Unlike veins, sinuses do not have valves. They also do not have a muscular layer; they are instead held together by the dura mater. The deoxygenated blood from the dural sinuses drains into the internal jugular veins before returning to circulation [1].

Dural venous sinus thrombosis (DVST) is a blood clot that has occluded one or more sinuses within the cerebral venous network. The annual incidence of dural venous sinus thrombosis is estimated to be three to five cases per million in adults [2] and comprises less than 1% of all strokes [3,4]. The incidence is slightly higher in pediatric patients, closer to seven cases per million, and the rate is highly increased in neonatal populations [5]. Pursuing this further, pediatric populations were found to have more non-obstructive thromboses, while adults had a higher rate of fully occlusive DVST [6].

Although there are copious predisposing factors, the core principles causing increased risk revolve around Virchow’s triad: hypercoagulability, stasis, and endothelial damage. In North America specifically, 19% of all patients (adult and pediatric) with DVST were found to have some form of hypercoagulable state [3], which can be secondary to various causes. For example, hereditary hypercoagulability is often secondary to mutations involving factor 5 Leiden, factor C and S, antithrombin, and prothrombin. Furthermore, increased estrogen causes hypercoagulability in adults, as it increases the levels of plasma fibrinogen and the activity of various coagulation factors within the body. Thus, not surprisingly, there is a correlation between thrombosis formation and female sex. One previous study found that 80% of patients with DVST were childbearing-age women [3], while another found that 65% of women who had developed DVST had factors that included pregnancy, contraceptive use, or hormonal replacement therapy [2]. Trauma is also a significant risk factor for development, as it can cause compression of the sinuses and stasis of blood flow, leading to thrombus formation [3]. Around 14% of patients with DVST were found to have head trauma [2], and that percentage increases to 35% if the patient has a skull fracture over a dural sinus [6]. This remains a predisposing factor in the pediatric population as well as adults; a previous case series saw that four out of five children with head trauma developed thrombi in the sigmoid sinus [7]. Infection is another risk factor seen in about 7% of patients with DVST in all populations. Additionally, brain malignancies were seen in 8% of DVST patients [1]. Approximately 25-30% of all DVSTs have no clear etiology [3].

The symptoms of DVST are caused by the blockage of the arachnoid granulations (also known as Pacchionian granulations) [5], which allow cerebrospinal fluid to enter the venous system. Consequently, the obstruction of this flow causes clinical signs indicative of elevated intracranial hypertension, with headaches being the most common symptom reported. This occurs in 80% of patients [2]. Although classically associated with subarachnoid hemorrhage, 10% of those with headaches experience what is characterized as rapid-onset, thunderclap pain [5]. About 25% of the time, patients with DVST have headaches with no other symptoms. Seizures are present in 40% of patients, followed by papilledema, which occurs about 28% of the time [2].

Due to the often nonspecific clinical presentation, the diagnosis of DVST requires imaging. Non-contrast computed tomography (CT) can be used acutely to exclude hemorrhage while concurrently diagnosing and/or ruling out other risk factors such as brain cancers, fractures, or abscesses [5]. A common clue to the diagnosis of non-contrast CT is an increased density within the thrombosed venous sinus or cortical vein if acute, commonly referred to as the “cord” sign [8]. Meanwhile, on contrast-enhanced CT, a filling defect will be visualized and is referred to as the “empty delta” sign when present within the superior sagittal sinus [8,9]. Furthermore, contrast-enhanced CT venography (CTV) can be used to localize the lesion more accurately and serve as a more sensitive alternative, as a previous study found that initial non-contrast CT scans can miss 30% of all DVST [2]. Magnetic resonance imaging (MRI) is another alternative, as it is more sensitive for detecting DVST as compared to non-contrast CT. Like CT, the appearance will vary depending on the acuity. As nonspecific symptoms such as headaches can delay imaging, MRI can be utilized to identify subacute or chronic thromboses. It is also helpful for determining resolution months later and is a valuable option to reduce the accumulation of radiation exposure over time. In the first week of formation, DVST appears isointense on T1-weighted images and hypointense on T2-weighted images. During the second week and onward, the lesion should appear hyperintense on T2-weighted images [2]. A “brush” sign may also be seen on susceptibility-weighted MR sequences when the presence of thrombosis drops the signal [8]. 

Treatment of DVST is primarily with anticoagulation medication along with treating any underlying causes of thrombosis, if applicable. Surgical intervention has resulted in poorer outcomes than anticoagulation [4,10]. Many patients who present acutely with no known predisposing factors are usually placed on long-term antithrombotic medication while hematologic and oncologic workups are being done.

## Case presentation

We report a case of a 16-year-old male with no relevant past medical history or surgical history. Notably, he was a nonsmoker and nondrinker and had no family history of malignancy. This adolescent presented to the emergency department two weeks after a combat sports competition where he sustained a traumatic injury to the head twice during a match. He was asymptomatic at the time of the injury, but several days later, he began developing a mild headache and blurry vision in his left eye.

The patient initially presented to his local urgent care center, where he reported a mild headache. At that time he was provided with a migraine cocktail containing butalbital, acetaminophen, and caffeine with instructions to go to the emergency department (ED) if the pain got worse. Later that evening, the patient developed photophobia and increased pain, did as instructed, and received an additional migraine cocktail (containing the same components as above) upon presentation to his local ED. His headache resolved, and the patient became asymptomatic with subsequent discharge and a recommendation to obtain a non-emergent, non-contrast MRI. He returned to the ED two days later with a painful headache, blurry vision, and non-bloody, nonbilious emesis, treated initially with another migraine cocktail. While attempting to obtain informed consent to do an emergent CT, the patient and his mother decided to wait for the MRI scheduled a few days later due to concerns regarding the risk of radiation exposure.

**Figure 1:**
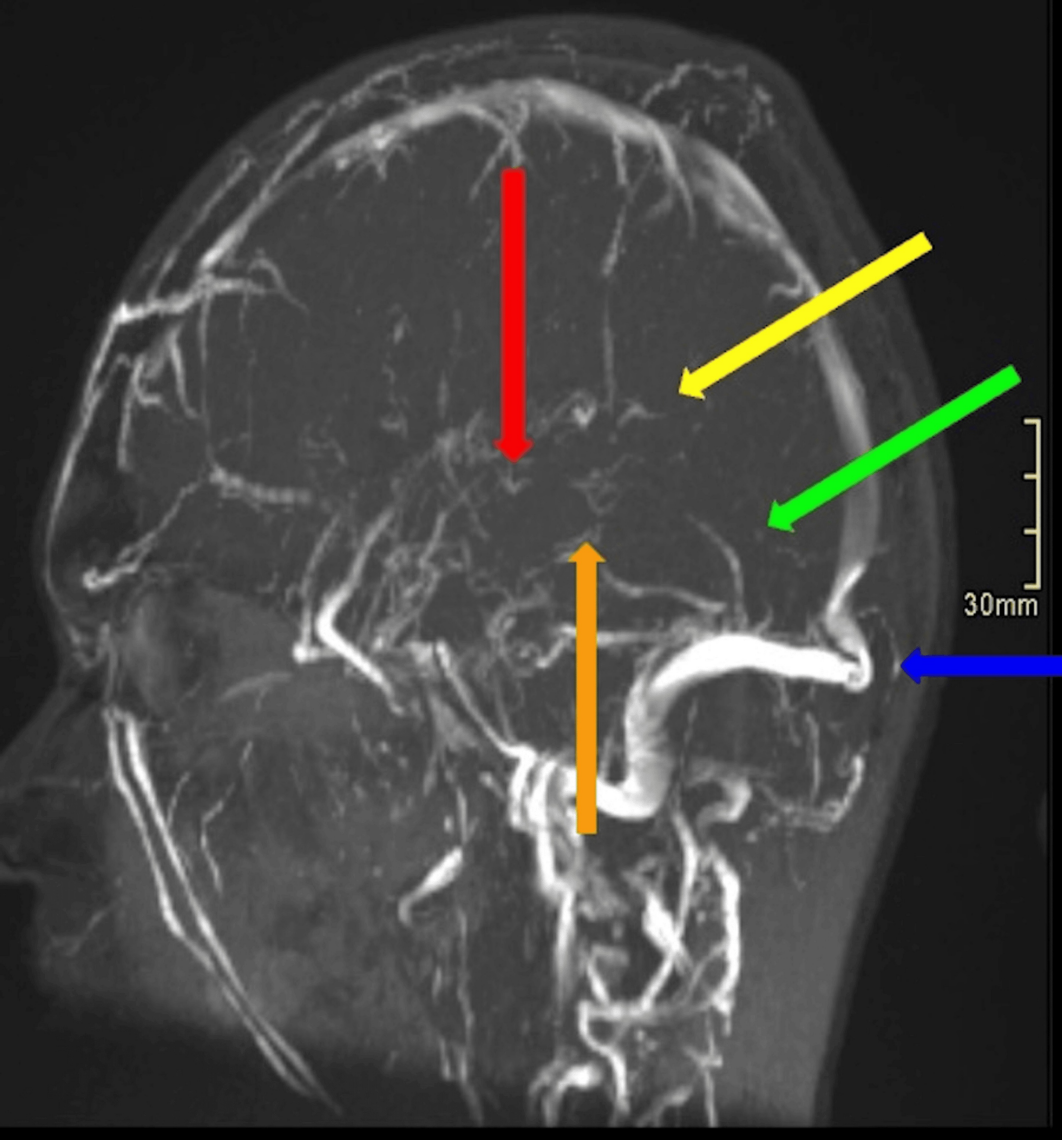
Volume-rendered sagittal view of the contrast-enhanced brain MRV. Displays filling defects involving the internal cerebral vein (red arrow), a vein of Galen (orange arrow), inferior sagittal sinus (yellow arrow), straight sinus (green arrow), and torcular herophili (blue arrow). MRV: magnetic resonance venography

**Figure 2:**
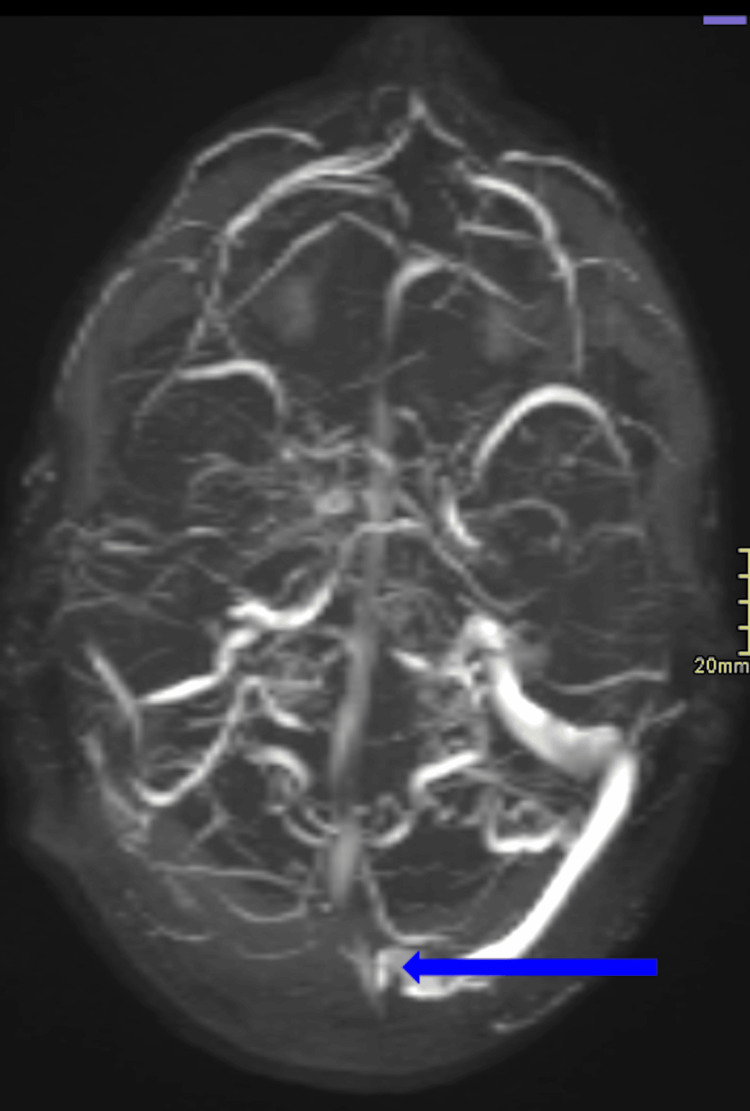
Volume-rendered axial view of the contrast-enhanced brain MRV. Displays a notable filling defect involving the superior sagittal sinus (blue arrow). The right transverse sinus is not well visualized, which may be secondary to significant hypoplasia, aplasia, or thrombosis. MRV: magnetic resonance venography

**Figure 3:**
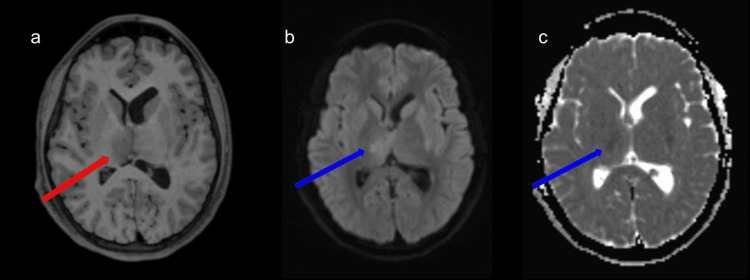
Concurrent brain MRI (with and without contrast). Axial views of T1-weighted (a), diffusion-weighted imaging (b), and apparent diffusion coefficient (c) sequences of the contrast-enhanced brain MRI display a T1 hypointense right thalamus (red arrow) with corresponding true restricted diffusion (blue arrows). This is consistent with a right thalamic infarct secondary to deep cerebral vein thrombosis.

**Figure 4:**
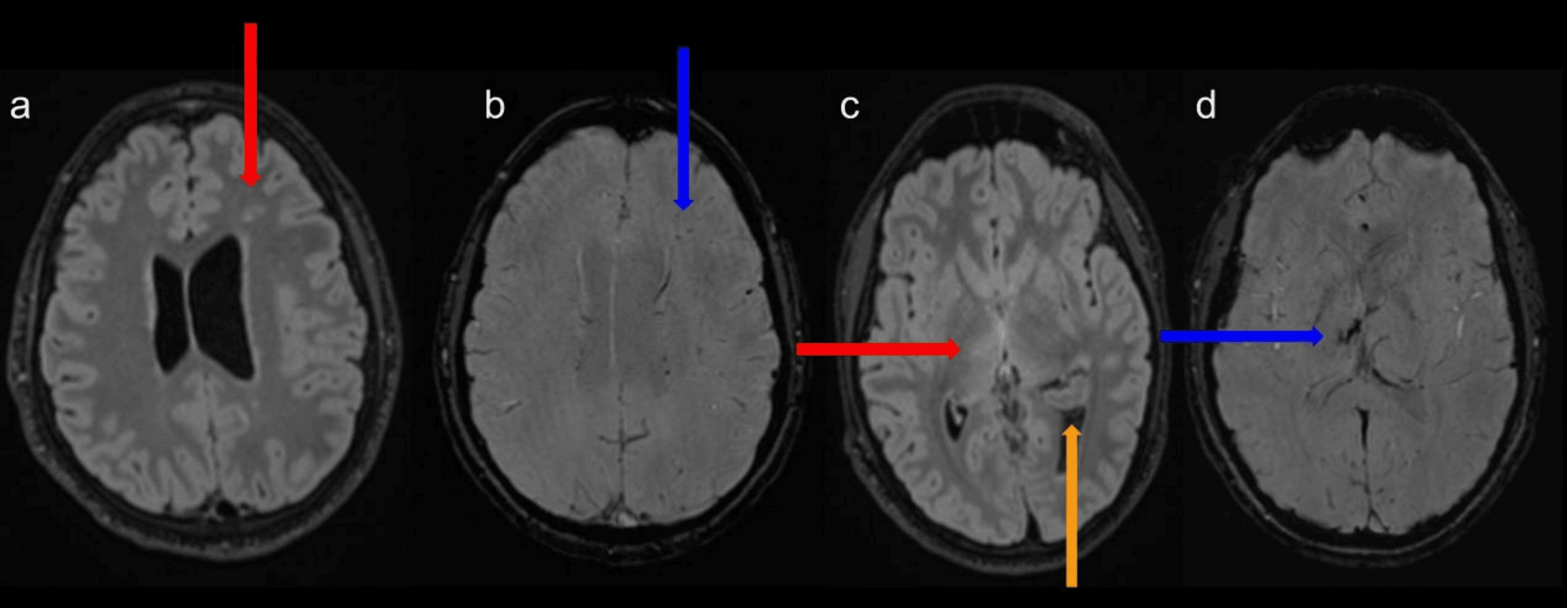
Axial views of T2-weighted and susceptibility-weighted imaging sequences of the contrast-enhanced brain MRI. Displays T2 hyperintensities within the right thalamocapsular region and left frontal lobe (red arrows) with corresponding susceptibility on susceptibility-weighted imaging (blue arrows). This is consistent with subacute hemorrhages/microhemorrhages given the clinical history. The left intraventricular hemorrhage (orange arrow) is also partially visualized.

Several days later, the outpatient MRI without contrast was completed, which displayed acute infarcts involving the bilateral basal ganglia and right thalamocapsular parenchyma, as well as abnormal susceptibility weighted imaging (SWI) hypointense signals within several deep veins and venous sinuses. The optic nerve sheath complexes showed signs of papilledema. The patient was called back to the emergency department, at which time he reported generalized weakness, dizziness, and decreased appetite, as well as displayed unsteady ambulation. An urgent MRA/MRV was ordered, which better displayed thromboses involving the internal cerebral veins, inferior sagittal sinus, vein of Galen, straight sinus, and torcula with partial extension into the bilateral transverse sinuses and the inferior aspect of the superior sagittal sinus (Figures 1, 2). A concurrent brain MRI (with and without contrast) displayed a T1 hypointense right thalamus, consistent with a right thalamic infarction secondary to deep cerebral venous thromboses (Figure 3). Additionally, subacute hemorrhages/microhemorrhages were present within the right thalamus and left frontal lobe, as well as an interval development of left lateral intraventricular hemorrhage (Figure 4). 

**Figure 5:**
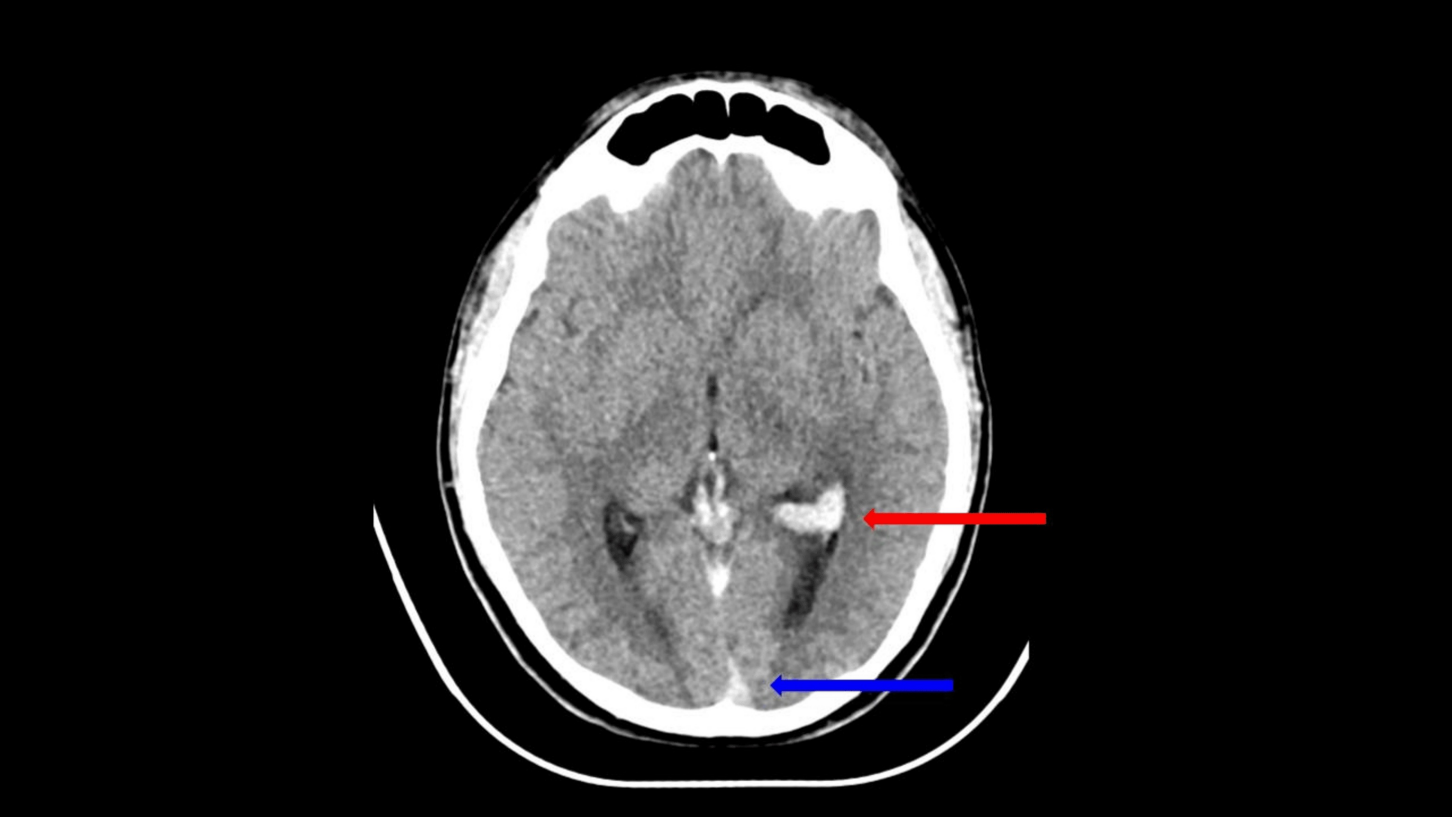
Axial view of the non-contrast head CT. Illustrates the "cord" sign within the inferior aspect of the superior sagittal sinus (blue arrow), indicative of thrombosis. Hyperdense blood products within the atrium of the left lateral ventricle are also partially visualized (red arrow).

**Figure 6:**
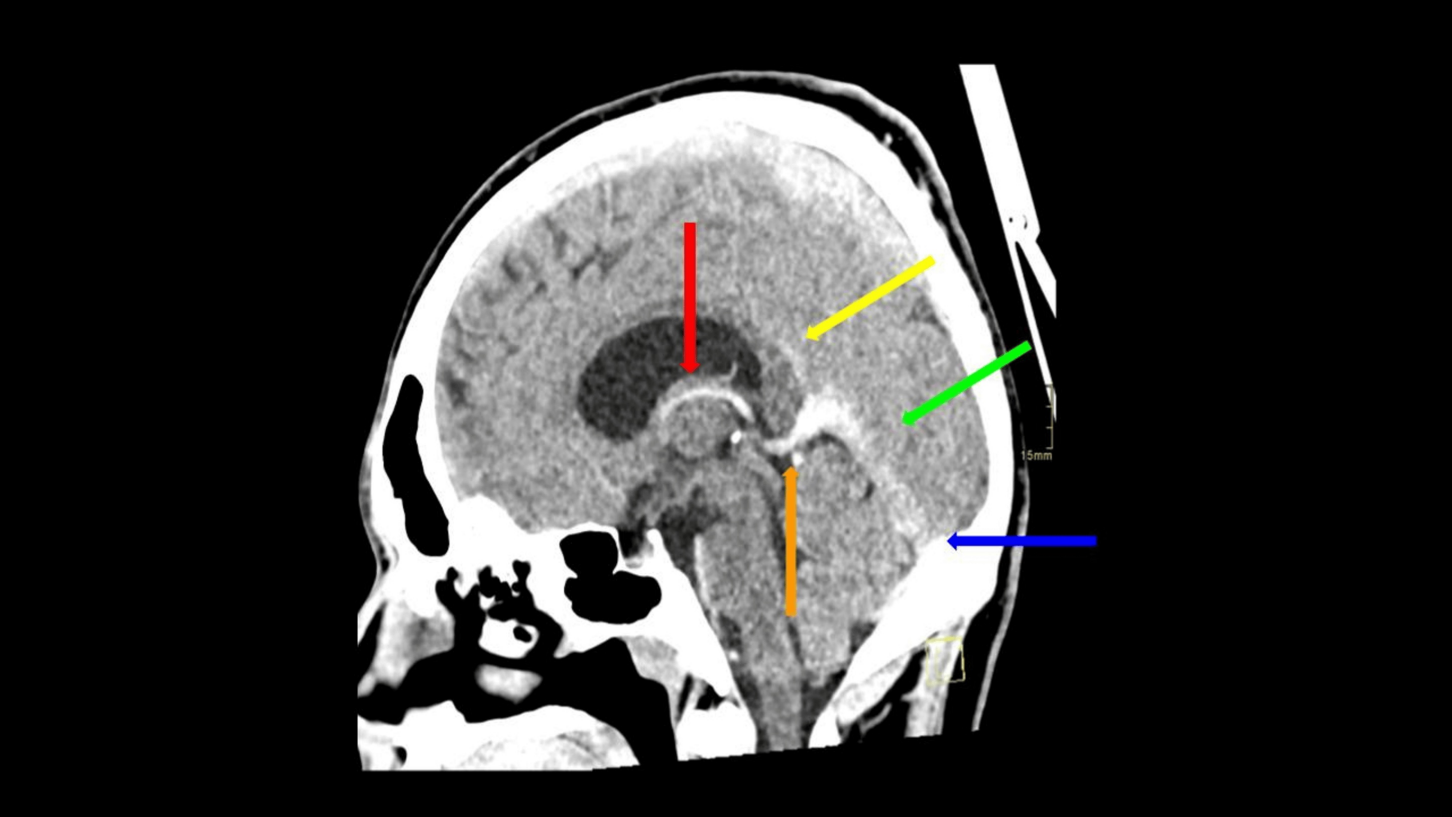
Sagittal view of the non-contrast head CT. Displays multifocal hyperdense venous contents compatible with DVST. The visualized occluded vessels include the following: internal cerebral vein (red arrow), a vein of Galen (orange arrow), inferior sagittal sinus (yellow arrow), straight sinus (green arrow), and torcular herophili (blue arrow).

The patient was treated with heparin therapy rather than a neurosurgical intervention. He was also evaluated by ophthalmology and found to have no clinical signs of papilledema, although he did have an intermittent disconjugate gaze. A follow-up CT was ordered the following day, which confirmed the hemorrhage involving the choroid plexus within the atrium of the left lateral ventricle and redemonstrated the dural venous sinus thromboses (Figures 5, 6). The patient continued IV heparin with rapid improvement of symptoms, later transitioned to enoxaparin, and was discharged shortly after.

**Figure 7:**
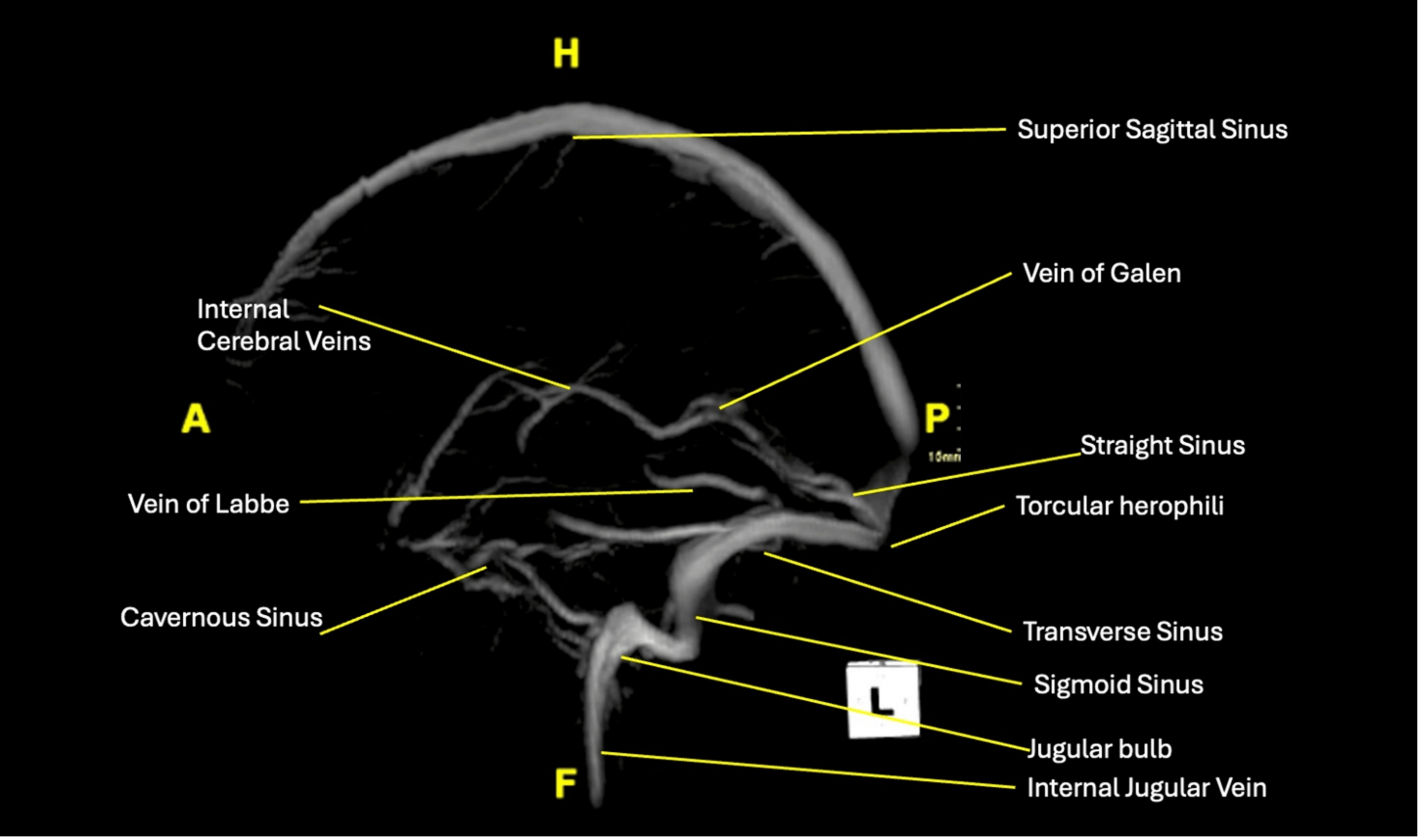
Volume-rendered sagittal view of the contrast-enhanced brain MR venography completed at the patient’s one-month follow-up after hospital discharge. Displays interval recanalization of the internal cerebral veins as well as partial recanalization of the vein of Galen, straight sinus, and torcula.

At his follow-up evaluation one month later, the patient had no laboratory studies to indicate a predisposition to thrombophilia and no longer exhibited any neurologic or intracranial bleeding symptoms. A brain MRI (with and without contrast) was repeated, which showed evolutionary changes in the right thalamic infarct and the resolution of the intraventricular hemorrhage (Figure 7). Furthermore, it displayed interval recanalization of the internal cerebral veins as well as partial recanalization of the vein of Galen, straight sinus, and torcular herophili. The patient continued enoxaparin therapy for another month, and repeat coagulation studies were normal.

## Discussion

This clinical vignette demonstrates the need for urgent imaging and timely radiologic interpretations. Prior to getting an MRI, this patient was evaluated by three providers, with each misdiagnosing the thromboses as an uncomplicated headache. Based on the American College of Radiology’s Appropriateness Criteria for Headaches (2019), in the setting of subacute trauma and neurological deficits (in our case, our patient had blurred vision and unsteady gait), the ACR reports CT and MRI as usually appropriate [11]. Furthermore, the ACR has appropriateness criteria specifically for head trauma, which states that new or progressive neurological deficits post-head trauma are highly recommended clinical indications for CT evaluation [12]. Even in the absence of thrombosis, MRI may have proved valuable at the time of initial diagnosis, as MRI can show diffuse axonal injury in the setting of mild head trauma or concussion [12].

Also, the patient and his family were persuaded to wait for their outpatient MRI instead of getting the emergent CT head due to the risk of radiation exposure in pediatric populations. Meanwhile, though, an argument could be made that the CT head was necessary due to his progressive clinical symptoms in the setting of recent head trauma, as this could have demonstrated intracranial hemorrhage earlier. Radiation exposure in children is associated with an increased risk for malignancy in about one in 5000 children who get a head CT [13], and the risk of radiation decreases with increasing age. In our case, the patient was 16 years old, further strengthening our argument regarding the need for urgent CT.

Moreover, this vignette is remarkable given the imaging characteristics and distribution of the radiologic findings. Our patient had developed extensive thromboses involving the internal cerebral veins, inferior and superior sagittal sinuses, a vein of Galen, straight sinus, and bilateral transverse sinuses. Studies have found the transverse sinus to be the most likely sinus to be occluded 86% of the time [1]. Additional studies found that when a DVST occurs in the superior sagittal sinus, hemorrhage occurs 60% of the time [14]. While this proved true in our case, the sizable thromboses extended into the internal cerebral veins, resulting in a unilateral thalamic infarct with associated petechial hemorrhage as well as an intraventricular hemorrhage, which is extremely rare relative to other intracranial hemorrhages.

## Conclusions

This case serves as a classic example of returning to the principles of coagulation based on Virchow’s triad of hypercoagulability, stasis, and endothelial damage. In our patient, with negative coagulation studies and a recent head injury, we can conclude that the trauma from the sports competition was the primary contributor to his thromboses. This case also highlights the importance of “red flags” that serve as indications for additional imaging. Finally, although delayed, the radiologic examinations were performed, and the patient was able to make a swift recovery after the appropriate diagnosis and treatment. At his follow-up evaluation months later, the patient was asymptomatic.
